# The complexities of investigating mitochondria dynamics in multiple sclerosis and mouse models of MS

**DOI:** 10.3389/fnins.2023.1144896

**Published:** 2023-07-25

**Authors:** Kelley C. Atkinson, Marvellous Osunde, Seema K. Tiwari-Woodruff

**Affiliations:** Division of Biomedical Sciences, School of Medicine, University of California, Riverside, Riverside, CA, United States

**Keywords:** multiple sclerosis, cuprizone, mitochondria, myelin, remyelination, demyelination, inflammation, EAE

## Abstract

Multiple sclerosis (MS) is a demyelinating, degenerating disorder of the central nervous system (CNS) that is accompanied by mitochondria energy production failure. A loss of myelin paired with a deficit in energy production can contribute to further neurodegeneration and disability in patients in MS. Mitochondria are essential organelles that produce adenosine triphosphate (ATP) via oxidative phosphorylation in all cells in the CNS, including neurons, oligodendrocytes, astrocytes, and immune cells. In the context of demyelinating diseases, mitochondria have been shown to alter their morphology and undergo an initial increase in metabolic demand. This is followed by mitochondrial respiratory chain deficiency and abnormalities in mitochondrial transport that contribute to progressive neurodegeneration and irreversible disability. The current methodologies to study mitochondria are limiting and are capable of providing only a partial snapshot of the true mitochondria activity at a particular timepoint during disease. Mitochondrial functional studies are mostly performed in cell culture or whole brain tissue, which prevents understanding of mitochondrial pathology in distinct cell types *in vivo*. A true understanding of cell-specific mitochondrial pathophysiology of MS in mouse models is required. Cell-specific mitochondria morphology, mitochondria motility, and ATP production studies in animal models of MS will help us understand the role of mitochondria in the normal and diseased CNS. In this review, we present currently used methods to investigate mitochondria function in MS mouse models and discuss the current advantages and caveats with using each technique. In addition, we present recently developed mitochondria transgenic mouse lines expressing Cre under the control of CNS specific promoters to relate mitochondria to disease *in vivo*.

## Introduction

Demyelination paired with inflammation contributes to neurodegeneration and disability in multiple sclerosis (MS), an autoimmune disease of the central nervous system (CNS) that affects 2.3 million people worldwide (Browne et al., 2014). While several elements are known to contribute to the neurodegeneration observed in MS, one factor that has been evident in MS, as well as other neurodegenerative diseases, is mitochondria dysfunction. In the context of MS, the loss of myelin contributes to a loss in saltatory conduction, resulting in an increased energy demand needed for the axon to maintain its resting membrane potential (Shepherd, 1988; Waxman and Black, 1995; Kaplan et al., 1997). The CNS has an extremely highly metabolic rate and consumes about 20% of oxygen at rest while only accounting for 2% of total body weight (Sokoloff et al., 1977; Silver and Erecińska, 1998), meaning even slight changes in energy demand could be deleterious for the CNS as a whole.

In this review, we will touch upon structure and function of mitochondria, discuss how mitochondria are currently studied in MS and in mouse models of MS, and how the current methods may only give a partial explanation of the pathophysiology of MS. Additional information on developments of metabolic pathway dysfunction in mitochondria and diseases can be found in other reviews (Frazier et al., 2019; Saneto, 2020; Moos et al., 2021; Baker et al., 2022). Later, we will discuss recently developed methodologies to assess mitochondria specific RNA and protein changes and development of mitochondria transgenic mouse lines that utilize inducible Cre recombinases in specific CNS cell types to study mitochondria. These recent methodologies will allow us to assess mitochondrial pathology in a cell-type specific manner and better understand contributions to neurodegeneration. Understanding how demyelination affects mitochondrial dysfunction and neurodegeneration will help us identify targets that can be used to develop novel therapeutics, provide neuroprotection, and improve the quality of life in patients with MS.

### Mitochondria

The mitochondrial proteome, around 1,200 proteins in humans, is under dual genomic control with 99% of proteins encoded by the nuclear genome. Mitochondrial (mt) DNA encodes 13 polypeptides and features 4 of the 5 respiratory chain complexes (Taylor and Turnbull, 2005; Islam, 2017; Rath et al., 2021) and is inherited maternally (Youle and Van Der Bliek, 2012). Notably, this mitochondrial genome is not protected by histones, making its mutation rate higher than nuclear DNA (Islam, 2017). Human mtDNA is a circular molecule of 16,569 base pairs that encodes the rRNAs and tRNAs necessary to support intramitochondrial protein synthesis using its own genetic code (Lin and Beal, 2006). Mitochondria contain multiple copies of mtDNA, and this multi-copy nature is explained by heteroplasmy- where both mutated and wildtype mtDNA coexist in the same cell (Fetterman and Ballinger, 2019; Rath et al., 2021). These proteins are synthesized in the cytosol, targeted, and then imported into the mitochondria in a coordinated and regulated manner. When the mitochondrial function is disrupted, specific quality control mechanisms are activated to maintain protein and organelle quality, which can also result in changes in gene expression or changes in the cell proteome. These mechanisms help to maintain cellular and organismal health by ensuring that the mitochondria function properly. The current understanding of how these different quality control mechanisms are integrated and maintain protein and organellar quality relevant for cellular and organismal health has been recently reviewed (Ng et al., 2021).

**Figure 1:**
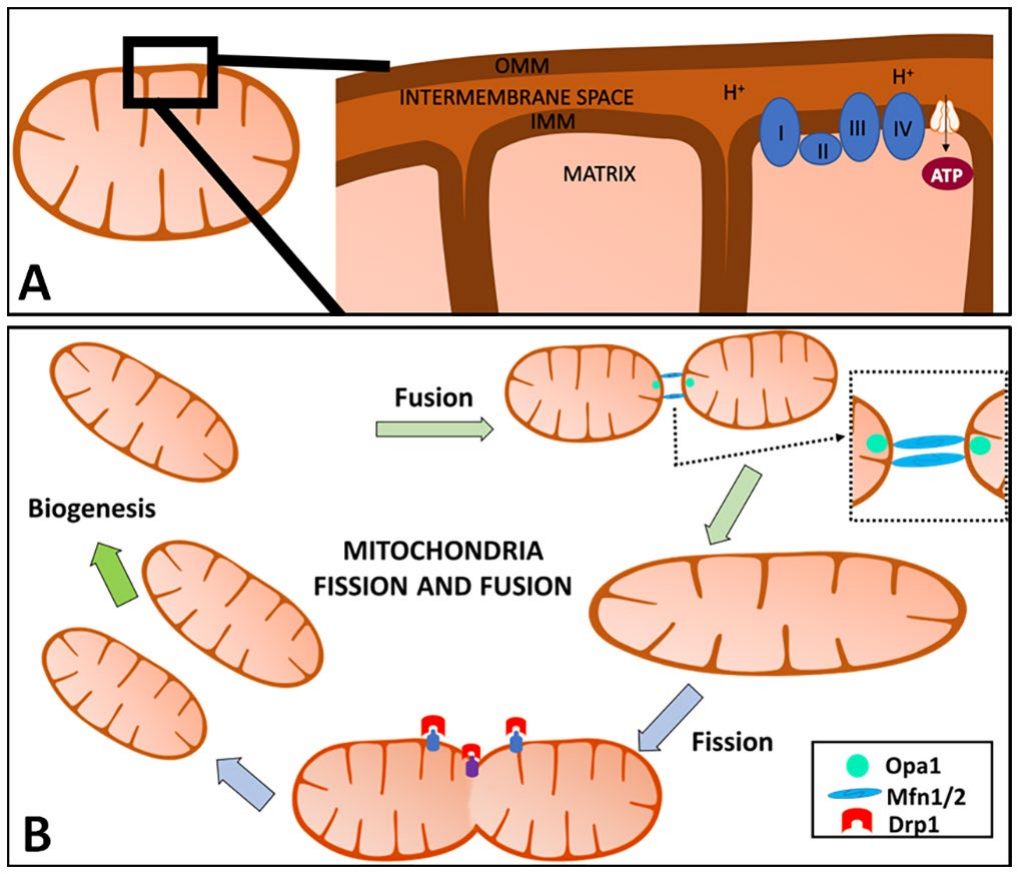
Overview of mitochondria and mitochondrial fission and fusion. **(A)** Mitochondria have an outer mitochondrial membrane (OMM), inner mitochondrial membrane (IMM), and the matrix **(A)**. The IMM is where the electron transport chain (ETC) is located and where adenosine triphosphate (ATP) production occurs. The ETC pumps protons from the mitochondrial matrix into the intermembrane space. This creates an electrochemical proton gradient across the IMM, providing ATP in addition to helping maintain the mitochondria membrane potential (MMP; ΔΨ_m_). **(B)** Mitochondria are highly dynamic organelles that divide and fuse using fission and fusion, respectively. Mitochondrial fission is defined as the division of one mitochondrion into two daughter mitochondria, while fusion is the union of two mitochondria resulting in one mitochondrion. These two factors allow for this organelle to adapt to cell needs at any given time and are regulated by a variety of cellular pathways including proteolytic processing, ubiquitylation, SUMOylation, phosphorylation and dephosphorylation. When fusion or fission occurs, the proteins are catalyzed by guanosine triphosphate (GTP)ase enzymes. In mammals, mitochondrial fission is mediated and controlled by dynamin-related protein 1 (Drp1) and dynamin 2 (Dnm2). Fusion has two GTPases: Mfn1 and Mfn2 which control the OMM, while the IMM is controlled by optic atrophy 1 (OPA1) in mammals. Created with Motifolio Biology Bundle.

Mitochondria have an outer mitochondrial membrane (OMM), inner mitochondrial membrane (IMM), and matrix (Figure 1A). The mitochondrial electron transport chain (ETC) is located in the IMM and consists of five protein complexes: Complex I (NADH dehydrogenase), Complex II (succinate dehydrogenase), Complex III (ubiquinone: cytochrome c oxidoreductase), Complex IV (cytochrome c oxidase), and Complex V [adenosine triphosphate, (ATP) synthase] (Wallace, 1992; Elston et al., 1998; Cheng et al., 2010; Ross et al., 2013). The tricarboxylic acid (TCA) cycle, also known as the Krebs cycle, takes place in the mitochondrial matrix and generates nicotinic adenine dinucleotide (NAD)H and flavin adenine dinucleotide (FAD)H_2_, which are then used as electron donors in the ETC. The ETC pumps protons from the mitochondrial matrix into the intermembrane space. This creates an electrochemical proton gradient across the IMM, generating ATP in addition to maintaining the mitochondria membrane potential (MMP; ΔΨ_m_). Maintaining the MMP is essential. A low MMP is associated with limited ATP, low superoxide production, and mitochondria elimination via autophagy, while a high MMP boosts ATP synthesis and superoxide production (Klingenberg, 1980; Korshunov et al., 1997; Lambert and Brand, 2004; Chinopoulos et al., 2009; Geisler et al., 2010; Belenguer et al., 2019).

In addition to producing ATP, mitochondria have additional functions vital to cells such as regulating oxidative stress, intracellular Ca^2+^ signaling homeostasis, and steroid synthesis (Rottenberg and Scarpa, 1974; Giacomello et al., 2020). Ca^2+^ storage in mitochondria is involved in the regulation of ion homeostasis, cell signaling, and apoptosis (Glancy and Balaban, 2012). These are all critical for cells in the CNS, especially in neurons due to these cells’ high metabolic rate and increased sensitivity to oxidative damage (Kann and Kovács, 2007). Furthermore, neurons require ATP for proper execution of neurotransmission and plasticity (Bordone et al., 2019). Due to this, proper neuronal development and survival are highly dependent on mitochondria function.

Many of the normal mitochondrial functions appear to be altered in neurons and oligodendrocytes (OLs) of MS postmortem tissue and animal models of MS due to disease-induced changes in energy demand and energy production (Witte et al., 2009; Licht-Mayer et al., 2020). The initiating insult that leads to mitochondrial defects in MS is not clear. Immunohistochemistry (IHC) studies performed in postmortem brain tissue and oxygen consumption rate (OCR) assessment in human cell lines suggest that neuronal mitochondrial dysfunction in the MS cortex could be central to MS pathology, and may contribute to impaired myelination and axonal damage (Li et al., 2013). Witte et al. (2009) show an increase in mitochondrial numbers in astrocyte and axons in active MS lesions using IHC co-localization. A mouse model where mitochondrial DNA (mtDNA) double-strand breaks (DSBs) were specifically induced in myelinating OLs (PLP:mtPstI mice) caused demyelination, axonal injury, and CNS inflammation (Madsen et al., 2017).

### Mitochondrial fission/fusion

Mitochondrial dynamics is a broad term to describe how mitochondria fission and fusion regulate number, morphology, and positioning changes within a cell. Mitochondrial fission is defined as the division of one mitochondrion into two daughter mitochondria, while fusion is the union of two mitochondria resulting in one mitochondrion (Detmer and Chan, 2007; Chen and Chan, 2009; Garone et al., 2018). These two factors allow for this organelle to adapt to cell need at any given time and are regulated by a variety of cellular pathways including proteolytic processing, ubiquitylation, SUMOylation, phosphorylation and dephosphorylation (Van der Bliek et al., 2013).

When fusion or fission occurs, the proteins are catalyzed by guanosine triphosphate (GTP)ases enzymes. In mammals, dynamin-related protein 1 (Drp1) and dynamin 2 (Dnm2) mediate and control mitochondrial fission (Figure 1B; Chen and Chan, 2009; Westermann, 2010; Barcelos et al., 2019). Fission in mammals is dependent on the main regulator Drp1 which consists of 4 domains: the N-terminal GTPase domain, a middle assembly domain, a B/variable domain (Garone et al., 2018), and a C-terminal GTPase effector domain (GED) (Chang et al., 2010; Van der Bliek et al., 2013; Hu et al., 2017). Drp1-dependent mitochondrial fission consists of translocation of Drp1 to the OMM, subsequently higher-order assembly, GTP hydrolysis, and disassembly (Hu et al., 2017). Drp1’s ability to bind to receptors on the OMM is mediated by the central stalk of the middle assembly domain to form a complex of Drp1 oligomeric helices (Garone et al., 2018). These Drp1 complexes can move along the mitochondria tubule, inducing constriction and then fission (Westermann, 2010; Van der Bliek et al., 2013; Liu et al., 2020). Recruitment of Drp1 by mitochondrial fission factors (Mff) regulates the division by recruiting Drp1 that oligomers into ring-like structures with GTP hydrolysis, contributing to the conformational change and mitochondrial constrictions.

Mitochondria OMM fusion is controlled by two large GTPases, mitofusins 1 and 2 (Mfn1, Mfn2), and optic atrophy 1 (OPA1) controls mitochondria IMM fusion (Chen et al., 2010). This process is characterized by three steps which include the tethering of the two mitochondria, the docking of the two membranes (IMM and OMM), and fusion of the two OMM (Garone et al., 2018). When GTP binds to its domains, Mfn undergoes a conformational change that allows for mitochondria docking and an increase of membrane contact sites. The Mfns contain GTP-binding domains that anchor onto the OMM by a C-terminal transmembrane domain. The OMM of the opposing mitochondria gets tethered by the interaction of the GTPase domains of Mfns. Fusion of the OMM is driven by GTP hydrolysis and GTP-dependent oligomerization, which is responsible for inducing Mfn’s conformational changes, bringing the two membranes in contact with one another allowing for mitochondria docking. This change also increases the membrane contact sites. Following OMM fusion, OPA1 drives IMM fusion with the help of a mitochondria-specific lipid, cardiolipin (CL) (Liu et al., 2020). The interaction between OPA1-CL on either side of the membrane tethers the two inner membranes following OPA1-dependent GTP hydrolysis (Liesa et al., 2009). It is important to note that IMM fusion occurs downstream of OMM fusion. Mfn2 is said to be present in the endoplasmic reticulum (ER) and controls the tethering of the ER onto the mitochondria which is observed in the mitochondrial constriction of the fission process (Liu et al., 2020). Mfn2 has been shown to be protective against neurodegeneration (Ishihara et al., 2009). Too much mitochondrial fission will cause fragmented-like mitochondria that are elongated and thin, and too much mitochondrial fusion will cause a shortage in energy production in the cell (Youle and Van Der Bliek, 2012).

Cuprizone, {[oxalic acid bis(cyclohexylidene hydrazide)] (CPZ) a selective and sensitive copper-chelating agent} and isonicotinic acid (an antibacterial drug) derivatives can induce fusion of pre-existing mitochondria resulting in large or “mega” mitochondria in OLs and hepatocytes (Asano et al., 1978; Wakabayashi, 2002; Acs et al., 2013). CPZ diet induces demyelination of a subset of CNS white matter tracts and is used as a demyelinating mouse model of MS. Severe enlarged mitochondria are seen in OLs in animals that were fed with CPZ-diet (Acs and Komoly, 2012; Acs et al., 2013). This is accompanied by the presence of enlarged mitochondria with disruption of mitochondrial β-oxidation, which is also seen in drug-induced hepatic injury (Hoppel and Tandler, 1973; Ramachandran et al., 2018). Usually, megamitochondria have normal oxidative phosphorylation and are different than swollen mitochondria. Perturbations in mitochondrial fission and fusion disrupt normal energetics, mitochondria movement and overall function (Wakabayashi, 2002) leading to neurodegenerative disorders.

### Mitochondrial transport

Mitochondria numbers vary between cells, and depending on cell type, mitochondria localization may not be homogenous. Initially, during development, mitochondrial transport occurs in the anterograde and retrograde directions continuously, with only a few mitochondria pausing for extended periods of time in the axon. As the neurons age, only about 25–30% of the total mitochondria are motile. In a healthy neuron, 70% of mitochondria are stationary and are generally found in areas of high ATP consumption (Fabricius et al., 1993) and the remaining 30% of mitochondria are mobile moving at a speed of approximately 1 μm/s (Kiryu-Seo et al., 2010). Proper axon function requires mitochondria to be transported to areas of high demand.

Differences in neuronal mitochondria morphology, density, movement and velocity have previously been noted between axonal and dendritic sub compartments in primary cell cultures (Chang et al., 2006). In live imaging of cultured neurons, punctate mitochondria are observed in axons; whereas dendritic mitochondria are more tubular (Lewis et al., 2018; Faitg et al., 2021). More than 40–50% of neuronal mitochondria were actively moving as compared to 8% of OL and 20% of astrocyte mitochondria when observed in primary cell cultures (see Table 1 in Meyer and Rinholm, 2021). Using *in vivo* imaging in the primary visual cortex of young mice, decrease in mitochondrial motility was observed with increases in neuronal activity (Silva et al., 2021).

The energetic demand of OLs for the process of myelination is enormous. It has been estimated that the generation of 1 gram of myelin demands 3.3 × 10^23^ ATP molecules (Meyer and Rinholm, 2021). Developing OLs have a high mitochondrial metabolism and primary mitochondrial oxidative phosphorylation (OXPHOS) rate before and during myelination. In line with this, the presence of high density long, tubular mitochondria have been observed in OL progenitor cells (OPC)s and developing OLs (Tondera et al., 2009; Yazdankhah et al., 2020). In addition, mitochondria in OPCs and immature OLs are clustered close to the tips of the processes and display Ca^2+^ signals upon neuronal activity (Kirischuk et al., 1995). Live imaging in organotypic mouse brain slices revealed that OL mitochondria move along primary processes and can enter and move within the myelin sheath (Rinholm et al., 2016). The mitochondria location also ensures local ATP production at the site of myelin induction. The OL mitochondria have slower mobility, and a smaller fraction of mitochondria move as compared with neurons and astrocytes. Thus, mitochondria play a role in the differentiation and initiation of myelin formation.

In the axon, mitochondria are mostly stationary until they are regulated by intracellular signals (Hollenbeck and Saxton, 2005). Mitochondria are then delivered to regions of high demand by moving along microtubule and actin tracts with the assistance of kinesin and dynein proteins (Hollenbeck and Saxton, 2005). Anterograde axonal transport is largely mediated by kinesin superfamily motor proteins which hydrolyze ATP to generate motile forces to shift cargos along the axon via microtubule tracks (Brady, 1985). This type of transport is pertinent to axonal health, and the transport is required for organelles, lipids, and proteins (Barnett et al., 2016). Retrograde transport is largely mediated by dynein superfamily motor proteins and is important for degradation products that need to travel back toward the soma (Barnett et al., 2016). Once mitochondria are transported to their destination, they are immobilized with syntaphilin (snph), a static anchor specific for axonal mitochondria, to keep mitochondria stationary in the axon. Without snph mitochondria become mobile and move along microtubule tracts within the axon (Ohno et al., 2014). In addition, snph is required for the increase in axonal mitochondrial volume in myelinated axons (Ohno et al., 2014).

*In vivo* analysis of mitochondria numbers especially in deeper layers of the brain and transport has been difficult. In a subset of neurons, using surface 2-photon and confocal imaging, mitochondria moving continuously and fast was observed (Boldogh and Pon, 2007).

### Mitophagy

Changes in the intracellular environment due to aging or neurodegenerative diseases can result in disrupted ATP synthesis, excessive reactive oxygen species production, and release of pro-death proteins. This can result in activation of cell death pathways. The selective clearance of damaged mitochondria in cells during cell stress or nutrient starvation is induced by mitochondrial mitophagy (Kubli and Gustafsson, 2012). Mitophagy is the process by which mitochondria and their contents are ubiquitinated, engulfed, and removed through lysosome degradation. The OMM is imperative for mitophagy function (Yoo and Jung, 2018). There, mitophagy can either occur via a non-receptor or receptor mechanism (reviewed in Swerdlow and Wilkins, 2020). Non-receptor mediated mitophagy, or classical mitophagy, starts with the activation of PINK1, that leads to recruitment of ubiquitin and Parkin (Li et al., 2023). Parkin ubiquitinates and phosphorylates mitochondrial proteins such as voltage-dependent anion-selective channel (VDAC), MFN1/2, and Translocase of the outer mitochondrial membrane complex subunit 20 (TOMM20). This initiates receptor adaptor protein recruitment, which includes p62, calcium-binding and coiled-coil domain-containing protein 2 (NDP52), optic neuropathy inducing protein (OPTN), Tax1-binding protein 1 (TAX1BP1), neighbor of BRCA1 gene 1 protein (NBR1), and subsequent interaction with microtubule-associated protein 1A/1B-light chain 3 (LC3) to form the autophagosome. The facilitation of autophagosome maturation, closure, and lysosome fusion occurs by the class III phosphoinositide 3-kinase (Vps34) and autophagy related (Atg)5/12/16 complex. Receptor mediated mitophagy is initiated by ubiquitination and phosphorylation of mitochondrial receptor proteins BCL2 Interacting Protein 3 (BNIP3), ligand of BNIP3L (NIX), FUN14 Domain Containing 1 (FUNDC1), pro-autophagy factor (AMBRA1), Prohibitin 2 (PHB2), or (cardiolipin) which facilitates their interaction with LC3 and GABA type A receptor-associated protein GABARAP for autophagosome formation. Similarly, the facilitation of autophagosome maturation, closure, and lysosome fusion occurs by the Vps34 and Atg5/12/16 complex. Receptor mediated mitophagy leads to the elongation of and closure of phagophore membranes, resulting in engulfment of the mitochondria.

### Mitochondria related changes in MS biofluids

Studies to assess the status of metabolically active mitochondria *in vivo* have not been attempted in the human brain. The indirect status of brain mitochondria during MS has been assessed by investigating levels of mitochondrial proteins and gene expression from biofluids in MS patients. For example, peripheral blood mononuclear cells (PBMC)s of MS patients shows impaired mitochondrial redox status and deficient antioxidant capacity (Gonzalo et al., 2019; Dominguez-Mozo et al., 2022). Lymphocytes selected by flow cytometry from the PBMC pool of MS patients showed significant decreases in mitochondrial respiratory chain complexes indicating compromised mitochondrial function as compared to normal controls (Gonzalo et al., 2019). Patients with PPMS showed a significant increase in cerebrospinal fluid (CSF) mtDNA compared to non-inflammatory neurologic disease controls (Leurs et al., 2018). Impaired metabolic status in MS patients has been observed with increases in both autophagic and mitophagy markers correlated with the active phases of the disease and with circulating lactate levels in both the serum and CSF obtained from MS patients (Castellazzi et al., 2019; Hassanpour et al., 2020; Giorgi et al., 2021). Similar changes are observed in other neurodegenerative diseases (Parkinson’s, PD and Alzheimer’s, AD) (Giorgi et al., 2021). Studies to correlate mitochondrial function with the type of MS disease and progression are underway.

### Mitochondria changes in MS postmortem tissue

Mitochondrial proteins, mitochondrial enzyme activity, and mtDNA mutations have been measured using immunohistochemical (IHC) and biochemical techniques in postmortem MS tissue (Campbell and Mahad, 2011; Barcelos et al., 2019). While MS postmortem tissue has been reviewed extensively, MS patients display heterogeneity in their symptoms, lesions, and disease-modifying treatments, meaning conclusions from these studies can be vague and oversimplified (Campbell and Mahad, 2011; Barcelos et al., 2019). Postmortem delay of removing and processing brains can range from a few hours up to 24 h, making analyses with postmortem tissue very convoluted. A study revealed that ΔΨ and ATP-production in mitochondria isolated from human brains up to 8.5 h postmortem can be observed, but rates of ATP production decrease with longer postmortem intervals (Barksdale et al., 2010). Dutta et al. (2006) isolated mitochondria-enriched fractions from postmortem MS cortex and showed a decrease in mitochondrial electron transport gene expression. They found that Complexes I and III were decreased by 61 and 40%, respectively (compared with control), but no changes in Complex IV (Dutta et al., 2006). In summary mitochondrial dysfunction in the motor cortex of MS patients was correlated to dysfunction of specific genes and not due to a deficiency in the number of mitochondria themselves (Dutta et al., 2006). Mitochondria findings from human MS postmortem tissue are summarized in Table 1.

**Table 1:** Mitochondrial changes in human MS postmortem tissue summary.

Chronic active MS lesions	Progressive MS	Subpial MS lesions	Inactive lesions
Increased porin in chronic active MS lesions compared with myelinated axons (Zambonin et al., 2011) No difference in axonal mitochondria content between RM and DM axons in RM regions close to DM areas in chronic MS lesions (Zambonin et al., 2011) Reduction in mitochondria content in shadow plaques compared with chronically DM axons in MS lesions (Zambonin et al., 2011) Decreased COXIV in axons in the rim of chronic active lesions (Mahad et al., 2009) Increased COXIV in MS lesions compared to control white matter and NAWM (Witte et al., 2009) Increased in mtHSP70 compared to control white matter and NAWM (Witte et al., 2009)	Respiratory deficient neurons (lacked COXIV but contained COXII) in DRG (Licht-Mayer et al., 2020) Respiratory deficient neurons also had increased mitochondria content, size, and number (Licht-Mayer et al., 2020) SPMS: individual neurons demonstrated decreased COXIV and mtDNA deletions throughout the gray matter (Campbell et al., 2011) SPMS: Decreased nuclear-encoded mitochondria ETC genes (COXI, COXIII, COXIV, COXV) (Dutta et al., 2006) PPMS/SPMS: Normal appearing gray matter showed reduced PGC-1α accompanied with neuronal loss in the cingulate gyrus (Witte et al., 2013)	Decreased COXIV in axons, astrocytes, and oligodendrocytes (Mahad et al., 2008)	Axons in inactive lesions had increased COXIV (Mahad et al., 2009)

Mitochondrial changes observed in different subtypes of MS, include chronic active MS lesions, progressive MS, pattern III MS lesions, and inactive lesions. Nearly all types of lesions in postmortem MS show changes in COXIV levels. DM = demyelinated, RM = remyelinated, NAWM = normal appearing white matter, DRG = dorsal root ganglia.

In chronic active MS lesions, IHC studies revealed increased mitochondria content (measured by porin) compared with myelinated axons. However, there was no difference in axonal mitochondrial content between remyelinated and demyelinated axons in remyelinated regions close to demyelinated areas in chronic MS lesions (Zambonin et al., 2011). In addition, a reduction in mitochondrial content was observed in shadow plaques compared with chronically demyelinated axons in MS lesions (Zambonin et al., 2011). Another study found respiratory deficient neurons that lacked COXIV but contained COXII that were prevalent within the dorsal root ganglia in progressive MS (Licht-Mayer et al., 2020). These respiratory deficient neurons also had increased mitochondria content, size, and number (Licht-Mayer et al., 2020).

A decreased COXIV expression in pattern III MS lesions in axons, astrocytes, and OLs has been observed by IHC (Mahad et al., 2008). Decreased COXIV activity was also observed in axons in the rim of chronic active lesions, while axons within inactive lesions displayed increased activity of COXIV (Mahad et al., 2009). However, another study found the opposite result, where COXIV activity was upregulated in MS lesions compared to control white matter and NAWM with an additional increase in mtHSP70, a mitochondrial stress protein (Witte et al., 2009). In addition, mitochondrial density in axons and astrocytes was increased in areas with active lesions compared to NAWM with a trend in inactive lesions (Witte et al., 2009).

The variation in COXIV expression between the groups may be due to the different types of lesions in both studies. Pattern III lesions (lesions located in the subpial layers of the cortex) are characterized by extensive OL apoptosis and hypoxia-like tissue injury (Lucchinetti et al., 2000). The MS postmortem tissue used in the study by Witte and colleagues did not have hypoxia-like damage or OL apoptosis. In addition, while Mahad and colleagues demonstrated an increase in COXIV activity in active lesions, they measured this particularly in axons, while Witte and colleagues measured COXIV activity in the entirety of the active lesion. In MS lesions, axonal COXIV activity may be decreased overall, however, total COXIV activity may be increased.

**Figure 2:**
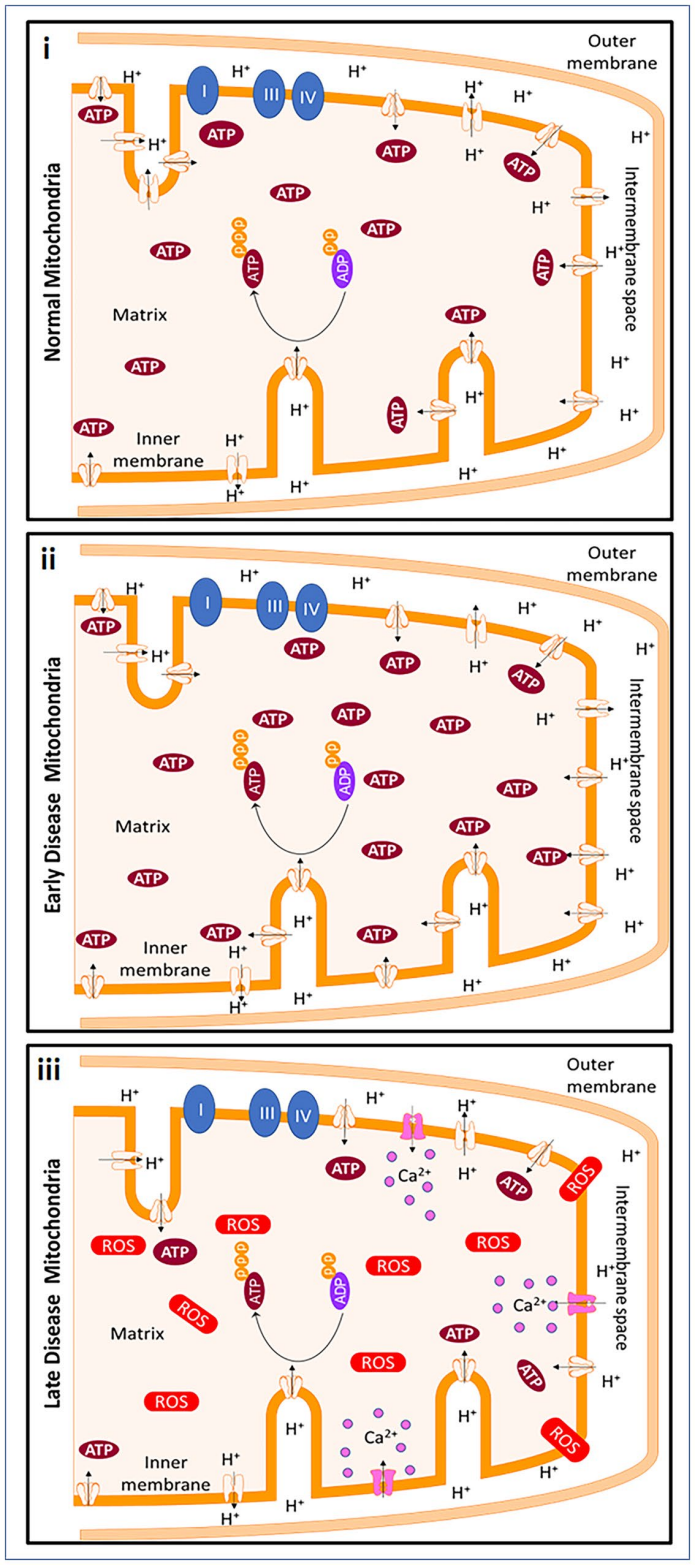
Mitochondrial changes in early MS and progressive MS. In homeostatic conditions, mitochondria produce ATP through ATP synthase and with the help of the protonmotive force of the ETC. ATP is produced due to the negative potential in the mitochondrial matrix when protons are pumped from the matrix into intermembrane space. Early in demyelinating disease, the loss of myelination causes a decrease of saltatory conduction in the axon contributing to an increase in energetic demand. To meet the new demands of the cell, ATP production by the cell is increased. However, the energetic demands cannot be met continuously by mitochondria. In addition, an increase in reactive oxygen species (ROS), causes ATP depletion. The Na^+^/Ca^2+^ exchanger ultimately reverses in an attempt to restore the polarization, but the calcium influx continues to be detrimental to the mitochondria and to the cells and leads to cell death. Created with Motifolio Biology Bundle.

An upregulation of snph was observed in tissues from progressive MS patients compared to healthy patients (Mahad et al., 2009). In homeostatic conditions, mitochondria produce ATP through the process of oxidative phosphorylation, using the proton gradient generated by the electron transport chain. In early MS, the loss of myelin can lead to an increase in the energetic demands of the affected cells, which can cause an increase in ATP production by the mitochondria. However, this increased demand is likely not sustainable in progressive MS, leading to depletion of ATP and an increase in reactive oxygen species (ROS). The Na^+^/Ca^2+^ exchanger in cells may attempt to restore the proton gradient, but this influx of calcium further damages the mitochondria and eventually leads to cell death. The timing of the disease will dictate the status of MS, which is hard to predict in postmortem brain tissue. Thus, the extent and timeline of all these studies are dependent on the type of MS and the course of disease the patient had during their lifetime (Figure 2).

Changes in mitochondrial proteins in MS postmortem tissue are supported by studies implicating mitochondria DNA (mtDNA) mutations and altered gene expression. These studies are discussed in Campbell and Mahad (2018) including the mtDNA nt13708A (Yu et al., 2008) and mtDNA T4216C (Andalib et al., 2016) variants. The importance of mtDNA for maintaining a healthy CNS is highlighted by a number of primary mtDNA disorders, where the entire nuclear DNA-encoded complex of mitochondrial respiratory chain, complex II, is spared (DiMauro and Schon, 2003; Zeviani and Di Donato, 2004). Neurons in postmortem secondary progressive (SP)MS tissue have also demonstrated mtDNA deletions and are respiratory deficient with decreased COXIV, which could be a major contributor to neurodegeneration. These changes were extensive in the gray matter (Campbell et al., 2011). In addition, specific mtDNA variants and changes in mtDNA copy numbers are also present in patients with MS (Blokhin et al., 2008; Vyshkina et al., 2008; Yu et al., 2008; Kenney et al., 2014a,b; Tranah et al., 2015). Furthermore, a subset of nuclear-encoded mitochondria ETC genes were decreased in the SPMS cortex compared to controls, including components for COXI, COXIII, COXIV, and ATP Synthase in upper motor neurons (Dutta et al., 2006). Whole-genome sequencing and replication studies in PPMS subjects (and not RRMS subjects) of European ancestry identified pathogenic variants: receptor expression-enhancing protein 1 (*REEP)1* and spastic paraplegia 7 (*SPG7*). These variants are also found in spastic paraplegia. REEP1 is widely expressed and localizes to mitochondria, whereas SPG7 encodes for paraplegin, a mitochondrial matrix protease embedded in the IMM (Atorino et al., 2003; Jia et al., 2018; Zheng et al., 2018). Using quantitative polymerase chain reaction (PCR) and western blot, reduced levels of peroxisome proliferator-activated receptor-gamma coactivator (PGC)-1α, a transcriptional coactivator and master regulator of mitochondrial function, were observed with neuronal loss in the cingulate gyrus and frontal cortex of normal appearing gray matter of MS patients, indicating that mitochondria dysfunction could be due partly to PGC-1α (Witte et al., 2013). One group was able to assess the mitochondria proteome in the MS prefrontal cortex using Surface-Enhanced Laser Desorption Ionization Time of Flight Mass Spectrometry (SELDI-TOF-MS) (Broadwater et al., 2011). The Cytochrome c Oxidase complex subunit (COX5B) expression with SELDI-TOF-MS demonstrated decreased COX5B expression in its mitochondrial fractions isolated from the cortex with western blot (Broadwater et al., 2011). This elegant study was not able to directly correlate the COX5B changes to mitochondrial changes of specific cells, as the study was performed from prefrontal cortex tissue. Mitochondria studies from postmortem MS tissue has been useful, however, for a better understanding of mitochondrial dysfunction in MS, animal models of MS are being used.

### Altered mitochondrial dynamics in animal models of MS

Due to the complexity of mitochondrial dysfunction in MS, mouse models that demonstrate inflammation, demyelination, and neurodegeneration are necessary to gain a better understanding of mitochondria pathophysiology. There are several mouse models of MS that mimic various aspects of MS pathology (Burrows et al., 2019). Different mouse models of MS such as experimental autoimmune encephalomyelitis (EAE), CPZ, lysolecithin, and ethidium bromide have been used to discuss mitochondrial dynamics in MS (Burrows et al., 2019). Each model has caveats because of differences in inflammation and levels of demyelination.

EAE is one of the best models for understanding MS pathophysiology, as it incorporates primary inflammation, demyelination, and neurodegeneration (Craner et al., 2003; Mangiardi et al., 2011; Hasselmann et al., 2017; Sekyi et al., 2021). This model uses a myelin peptide to induce an autoimmune reaction that causes levels of motor paralysis, cognition, and visual deficits in affected mice and recapitulates symptoms seen in MS. Similar to MS, EAE mice can develop lesions anywhere in the CNS, causing variation between age and sex matched mice. An additional demyelinating model includes the CPZ diet-induced demyelination. While CPZ does not have peripheral inflammation, this mouse model has consistent demyelination in various white matter regions including the corpus callosum, cerebellum, and hippocampus in the brain, but not the spinal cord, after varying amounts of length on the diet (Matsushima and Morell, 2001; Kipp et al., 2009). In addition, when the CPZ diet is replaced by a normal diet after a few weeks of demyelination, spontaneous remyelination occurs, allowing the use of this model to study remyelination induced changes. While it is known CPZ is a copper chelator, the true mechanism of CPZ diet-induced demyelination is unknown. CPZ diet may lead to mitochondrial dysfunction through interruption of oxidative metabolism in OLs, which rely on oxidative metabolism for most of their energy supply and eventual OL apoptosis (Vega-Riquer et al., 2019). The chronic CPZ diet induces formation of megamitochondria in the liver tissue as well as on OLs after 3 weeks (Acs and Komoly, 2012). Mitochondria have also been shown to be enlarged after 6 weeks of cuprizone and in several mouse models of demyelination (Ineichen et al., 2020). Because copper is known to have important roles in the mitochondria ETC (Xu et al., 2013), using CPZ as a method to understand mitochondrial dynamics in the complex of MS disease may be too convoluted. Both models, in addition to other models of demyelination such as lysolecithin and ethidium bromide, have been used to understand mitochondrial dynamics in MS. Lysolecithin and ethidium bromide are injected stereotactically in the CNS and reproducibility of the location and the size of the lesion can also vary. There have been several mitochondrial studies with these mouse models that will be briefly discussed below and are also summarized in Table 2.

**Table 2:** Summary of altered mitochondrial dynamics in animal models or genetic models of demyelination.

	Experimental autoimmune encephalomyelitis	Cuprizone diet; 0.2% unless stated otherwise	Genetic models
Morphology	EAE Day 10: vacuolization and dissolution of mitochondrial cristae (Qi et al., 2006) EAE Day 16: fragmented and swollen mitochondria (Holman et al., 2020) Chronic EAE: no change in in spinal cord neurons (Rosenkranz et al., 2021) Altered morphology in OLs during chronic EAE (Steudler et al., 2022)	3 weeks: Megamitochondria in oligodendrocytes (Acs and Komoly, 2012) 5 weeks: Mitochondria swelling (Faizi et al., 2016) 6 weeks: Increased mitochondria size correlates with increased g-ratio (Luo et al., 2017)	PLP1-overexpressing model shows increased mitochondrial density (Hogan et al., 2009) Increased mitochondrial density in *Shi* model (Joshi et al., 2015) Snph knockout mice have shorter and thicker mitochondria compared to controls (Ohno et al., 2014)
Fission and fusion	Increased Drp1 in lesioned spinal cord at peak EAE disease (Luo et al., 2017) Drp1 peptide inhibitor was neuroprotective (Luo et al., 2017)	6 weeks: Increased Drp1 in the corpus callosum (Luo et al., 2017), inhibiting Drp1 was neuroprotective.	SLC25A46 knockout (OMM protein) demonstrated degenerating dendrites, enlarged mitochondria in Purkinje cells, decreased ATP production (Li et al., 2017) PLP4e mice have increased Mfn2 (Thai et al., 2019) Opa1,PLP mutants have increased mitochondrial size with genetically induced mitochondria function (Ineichen et al., 2020)
Transport	Syntaphilin deletion does not benefit clinical symptoms in EAE (Joshi et al., 2015)	6 weeks 0.3% cuprizone with rapamycin (10mg/kg): Syntaphilin immobilization facilitates the survival of demyelinated axons (Ohno et al., 2014) Demyelinated axons deficient in syntaphilin degenerated at a greater rate than wildtype axons (Ohno et al., 2014)	Snph knockout mice facilitated axon regenerative capacity (Zhou et al., 2016) Deletion of Snph in dysmyelinating *Shi* mice prolonged survival and reduced cerebellar degeneration (Joshi et al., 2015)
Mitochondrial polarization	Depolarized mitochondria in a RRMS EAE model (Sadeghian et al., 2016)	5 weeks: Depolarized MMP, Mitochondria swelling, Cytochrome c release (Faizi et al., 2016)	No studies found
Mitochondrial function (Seahorse)	No studies found	6 weeks 0.3% cuprizone: Decrease in maximal respiration (Singhal et al., 2020) 4–5 weeks of 0.2% cuprizone in water. Decrease in respiration (Patergnani et al., 2021)	No studies found
Mitochondria transcription factors	Mitochondrial gene transcripts suppressed in motor neurons during CNS inflammation in the EAE model (Schattling et al., 2019)	12 weeks: Decreased PGC-1α, NRM-1, TFAM, and increased Drp-1 mRNA levels (Shiri et al., 2021)	No studies found

While there is extensive research on mitochondrial fission, fusion and transport in genetic knockout models, there are few studies on mitochondrial dynamics with EAE or CPZ mouse models of MS.

Demyelination can directly affect mitochondria dynamics due to changes in fission and fusion (Ohno et al., 2014). Fission and fusion are critical to mitochondria because they not only affect mitochondria morphology, but also affect mitochondria distribution within the cell. Drp1 has been effectively studied in both the EAE and CPZ mouse models. Luo et al. observed increased Drp1 expression in the lesioned spinal cord at peak EAE disease and corpus callosum after 6 weeks on the CPZ diet (Luo et al., 2017). Hyper-activation of Drp1 in culture contributed to an increase in mitochondrial fission, however, inhibiting Drp1 activation had a neuroprotective effect in both EAE and CPZ (Luo et al., 2017). Direct manipulation of mitochondrial proteins by deleting solute carrier family 25 member (SLC25A)46, an OMM protein, showed degenerating dendrites, enlarged mitochondria in Purkinje cells, and decreased ATP production compared to controls (Li et al., 2017). In addition, there was an increase of Mfn2 expression in proteolipid protein (PLP)4e mice, a demyelinating mouse model containing extra copies of myelin genes (Thai et al., 2019). One study also showed decreased mitochondrial complex expression in EAE after depletion of LKB1 from astrocytes (Kalinin et al., 2020). In addition, a recent study using transgenic mice showed that neuronal overexpression of peroxisome proliferator-activated receptor gamma coactivator 1 (Ppargc1a) gene, which encodes PGC-1α, led to increased numbers of mitochondria, COXIV activity, and respiratory capacity. Neuronal deletion of Ppargc1a aggravated EAE-induced spinal cord neurodegeneration, while neuronal overexpression of Ppargc1a ameliorated it (Rosenkranz et al., 2021). While these studies show direct changes of mitochondria, these modifications are observed after protein knockout or gene overexpression. Due to the genetic and proteomic alterations, these studies are unable to translate to human disease and postmortem tissue.

Mitochondrial transport is altered in demyelinating mouse models (Andrews et al., 2006; Ohno et al., 2014; Joshi et al., 2015). Enhancing axonal mitochondrial transport in snph knockout (KO) mice was shown to facilitate axon regenerative capacity (Zhou et al., 2016). This was also observed in the dysmyelinating shiverer (*Shi*) mouse model that was crossed with a mouse lacking syntaphilin. *Shi* is often used as a mouse model for progressive MS and provides metabolic matching by increasing the axonal mitochondrial mass (Andrews et al., 2006). Deletion of snph in the *Shi* mouse model significantly prolonged survival and reduced cerebellar degeneration (Joshi et al., 2015). Interestingly, during CPZ demyelination mitochondria immobilization mediated by syntaphilin facilitates the survival of demyelinated axons (Ohno et al., 2014). Demyelinated axons that are also deficient in syntaphilin degenerated at a greater rate than wildtype axons (Ohno et al., 2014). However, deletion of snph did not have an effect in EAE (Joshi et al., 2015). All these studies show indirect evidence that snph engages in facilitating the survival of axons and preventing neurodegeneration.

### Mitochondria dynamics in other neurodegenerative diseases

As expected, modifications and aberrations of fission and fusion are associated with neurodegenerative diseases such as Autosomal Optic Nerve Atrophy, PD, AD, and Huntington’s Disease (HD) (Delettre et al., 2002; Dodson and Guo, 2007; Bossy-Wetzel et al., 2008; Chen and Chan, 2009). Since mitochondrial dysfunction is observed in other neurodegenerative diseases this could be a universal mechanism of neurodegeneration.

Autosomal Optic Nerve Atrophy and PD have demonstrated abnormalities in mitochondrial fission and fusion. Opa1 mutations are reported to be responsible for Autosomal Optic Nerve Atrophy and have been reported to be associated with MS-like disorders in patients (Yu-Wai-Man et al., 2016) based on the McDonald criteria for MS diagnosis. In addition, mutations in Mfn2 caused segmental axonal degeneration without cell body death (Misko et al., 2012). Two genes involved in hereditary PD are PINK1 and Parkin, which are both important in mitochondria integrity (Dodson and Guo, 2007). Mutation or loss of Parkin and PINK1 in human SH-SY5Y cells resulted in exacerbated mitochondrial fragmentation that is mediated by Drp1 (Filichia et al., 2016; Liu et al., 2020). Interestingly, a statistically significant increase in PINK1 and Parkin levels in the CSF paired with serum samples of patients with relapsing remitting (RR)MS in the acute phase highlight the importance of mitophagy in the etiopathogenesis and progression of MS (Cossu et al., 2021). AD postmortem brains have shown abnormalities in mitochondria structure (Baloyannis, 2006; Bossy-Wetzel et al., 2008; Wang et al., 2008, 2009; Chen and Chan, 2009) similar to that seen in MS postmortem tissue (Li et al., 2013).

### The difficulty in assessing mitochondria with the available and published techniques

Most studies of mitochondrial dynamics rely on cultured cells, where mitochondria are imaged at a high resolution. However, *in vitro* studies do not completely represent what occurs in complex diseases like MS. In the recent past, detailed mitochondria studies were even difficult in animal models due to the unavailability of transgenic mice to specifically study mitochondria from distinct cell types. The recent availability of newer transgenic mice and techniques will allow more specific studies, however, there are still limitations to studying mitochondrial pathology. Here is a summary of several techniques used to probe mitochondrial dynamics within the context of MS disease. Table 3 lists techniques used to measure mitochondria activity *in vitro* and *in vivo.*

**Table 3:** Techniques used to study mitochondria.

*In vitro*	*Ex vivo*	*In vivo*
Transmission electron microscopy: mitochondria density, morphology, shape, and cristae complexity (Qi et al., 2006) Mitochondria membrane potential TMRM: Mitochondria function (Misko et al., 2012; Bénardais et al., 2013; Ren et al., 2016) Seahorse Analyzer: Mitochondria function (Licht-Mayer et al., 2020) Kymographs: Mitochondria transport (Kiryu-Seo et al., 2010; Zambonin et al., 2011; Misko et al., 2012) ATP probe: measures ATP and H_2_O_2_ production (Imamura et al., 2009) Mass spectrometry-based methods for analyzing the mitochondrial interactome in mammalian cells (Koshiba and Kosako, 2020; Gonzalez-Franquesa et al., 2021) Mitochondrial dysfunction induces serine dependency in cells and can be measured by 13C labeling to determine serine synthesis and LC-MS/MS quantitation of 12C and 13C serine in media (Bao et al., 2016)	Transmission electron microscopy: mitochondria density, morphology, shape, and cristae complexity (Errea et al., 2015) Three-dimensional electron microscopy: mitochondria morphology, size (Ohno et al., 2014) Mitochondria photoconversion lentivirus: Mitochondria trafficking and movement (Licht-Mayer et al., 2020) DsRed2 Lentivirus: Mitochondria visualization and movement (Ohno et al., 2014; Errea et al., 2015) Kymographs: Mitochondria transport (Ohno et al., 2014; Yin et al., 2016)	Transmission electron microscopy: mitochondria density, morphology, shape, and cristae complexity (Hogan et al., 2009; Nikić et al., 2011; Zambonin et al., 2011; Joshi et al., 2015; Ineichen et al., 2020) 3D electron microscopy: mitochondria morphology, size (Ohno et al., 2014; Yin et al., 2016) Two-photon intravital microscopy of spinal cord slices from ODCmitoGFP-Tomato animal (Steudler et al., 2022). Immunohistochemistry: Regional visualization of mitochondria (Hogan et al., 2009; Zambonin et al., 2011; Joshi et al., 2015; Honorat et al., 2017; Thai et al., 2019) Western Blot: Mitochondria protein expression (Joshi et al., 2015; Filichia et al., 2016; Ferro et al., 2017; Luo et al., 2017; Mancini et al., 2018; Singh et al., 2018; Ng et al., 2019; Thai et al., 2019; Djordjevic et al., 2020) DsRed2 Lentivirus: Mitochondria visualization and movement (Ohno et al., 2014; Errea et al., 2015) Mitochondria membrane potential: Mitochondria function (Sadeghian et al., 2016) Mitochondria membrane potential Mitotracker Red: Mitochondria function (Qi et al., 2006) ATP Assay (Yin et al., 2016) ATP probe: measures ATP and H_2_O_2_ production (Imamura et al., 2009; van Hameren et al., 2019) Seahorse Analyzer: Mitochondria function (Djordjevic et al., 2020; Singhal et al., 2020)

Mitochondrial dynamics in cells have been investigated *in vitro* using cell culture as it is easier to interpret results in one cell type. The studies that have been performed *in vivo* and *ex vivo* are geared toward specific regions of the CNS that are affected, i.e., spinal cord. Use of mitochondrial gene knockout models has given insight into the role of specific genes and proteins in mitochondrial dynamics globally and in specific cell types.

To visualize mitochondria density, morphology, shape, and cristae complexity in a specific region of the CNS, transmission electron microscopy (TEM) is often used. The demyelination model and timepoint after inducing demyelination contributes to a variety of results that have been previously published. In the caudal cerebellar peduncle in rats using ethidium bromide induced demyelination followed by remyelination, there was an increase in mitochondria density in remyelinated axons compared to demyelinated axons with EM, indicating a modulation of energy dynamics after remyelination (Zambonin et al., 2011). An increase in axonal mitochondrial density in the proteolipid protein (PLP)1-overexpressing mice compared to the normal mice was observed (Hogan et al., 2009). An increased density of mitochondria was also observed in Purkinje cell soma in the dysmyelinated *Shi* model (Joshi et al., 2015). One EAE study demonstrated vacuolization and dissolution of mitochondrial cristae evident in optic nerve mitochondria 10 days post induction (dpi) (Qi et al., 2006). Using a focal axon demyelinating model of MS, mitochondria appeared swollen with TEM 6 dpi (Nikić et al., 2011). Furthermore, with an acute oxidative stress model, axonal mitochondria were rounder with more complex cristae after acute oxidative stress 24 h after exposure (Errea et al., 2015). This study used extensive quantification to calculate mitochondrial circularity and roundness. Three-dimensional (3D) EM can be utilized as well to determine mitochondria morphology and size 3D. An increase in shorter and thicker mitochondria numbers was seen with 2D EM in the P_0_ CNS juxtaparanodal axon (Yin et al., 2016). Using 3D EM larger and longer mitochondria in demyelinated axons were observed, whereas snph-KO mice had shorter and thicker mitochondria compared to controls (Ohno et al., 2014). While TEM and 3D EM are advantageous to understanding mitochondrial morphology, one can only infer how the morphology relates to mitochondria dynamics and function.

Live slices imaged with two-photon microscopy using transgenic fluorescence mitochondria is another way to observe live mitochondria (Steudler et al., 2022). However, quantification is difficult due to the high fluorescence levels and decreased magnification compared to any EM study.

To observe mitochondria expression and function in distinct cell types IHC is still widely utilized. This allows visualization of mitochondria, but on much lower magnification compared with TEM and 3D EM. IHC allows for understanding of mitochondria content in specific cell types using colocalization of several antibodies to understand protein expression specific to regional mitochondria function. Both TOMM20 and Porin/VDAC are used as markers of mitochondria content and are quantified using ImageJ and puncta analysis (Zambonin et al., 2011; Honorat et al., 2017; Thai et al., 2019). These proteins can also be used to determine whether other proteins are localized to the mitochondria or the cell soma in culture (Honorat et al., 2017). Mitochondria IHC can determine ETC function by measuring OXPHOS subunits. COXI oxidizes NADH and is used as a marker to determine overall ETC function (Joshi et al., 2015). IHC can also measure mitochondria activity with antibodies for Cytochrome C oxidase or COXIV (Hogan et al., 2009). Studies that assessed mitochondria fusion using IHC in demyelinated optic nerves saw an increase in Mfn2 expression in a chronic progressive demyelinating mouse model (Thai et al., 2019).

Western blot analyses are used to measure the abundance of mitochondria-specific protein content in tissue homogenates. Homogenized tissue is typically an assortment of cell types in a region, so the changes in mitochondrial protein observed cannot be linked to a specific cell type or function. Some studies have used whole cell lysate, or isolated mitochondria from tissue blocks followed by Western blot, giving a more accurate picture of mitochondria protein function. Using Western blot analysis, an increase in Mfn2 expression in optic nerve lysates was observed in mice with extra copies of the PLP gene (PLP4e) (Thai et al., 2019). To understand mitochondria transport in the dysmyelinated *shi* mouse model, quantification of snph was beneficial (Joshi et al., 2015). EAE spinal cord lysates were used to measure ETC levels. The results showed an increase in COXI and III levels early in EAE disease (Ng et al., 2019). The change in mitochondria ETC content in EAE was difficult to explain and could not be attributed to activity by specific group of cells, as neurons, OLs, astrocytes and immune cells were all present in the lysate (Mancini et al., 2018; Singh et al., 2018). All the ETC complexes have often been measured by Western blot using an OXPHOS antibody cocktail, which contains subunits of each complex, COXI through ATP Synthase (Ferro et al., 2017; Djordjevic et al., 2020). This cocktail allows for visualization of COX expression levels individually on one blot to compare between groups. Fission and fusion are often measured with Western blot as well, using Drp1 for measurements of mitochondria fission and Mfn2 for mitochondria fusion. An increase in Drp1 expression in the mitochondria rich fraction was found in a PD mouse model (Filichia et al., 2016). Drp1 hyperactivation has been evident in both EAE and cuprizone-induced demyelination models (Joshi et al., 2015; Luo et al., 2017; Thai et al., 2019).

The use of dyes and lentiviruses to permeate mitochondria are routinely used to assess their location and movement. These can be used in both primary cell cultures and brain slices to observe live movement and transport of mitochondria (Errea et al., 2015; Licht-Mayer et al., 2020). Depending on the dye or the lentivirus used, it is possible to study mitochondria function regionally in an animal model of MS. One study used a lentivirus to tag mitochondria in the Purkinje cell layer of the cerebellum (Licht-Mayer et al., 2020). A lentivirus containing mitochondrial-targeted DsRed2 injected into slice cultures (Ohno et al., 2014; Errea et al., 2015) allowed mitochondria to be visualized after stimulation. This could help understand mitochondria transport and movement over a period of time in mouse models of MS. Kymographs are also used concurrently with DsRed2 as a method to understand mitochondria transport (Kiryu-Seo et al., 2010; Zambonin et al., 2011; Misko et al., 2012).

Another method to quantify mitochondria function is by measuring MMP. Tetramethylrhodamine methyl ester (TMRM) is a cationic, potentiometric dye that measures MMP (Sadeghian et al., 2016). When mitochondria are depolarized, the TMRM dye no longer remains in the mitochondria. Instead of using slice cultures with the DsRed2 lentivirus, TMRM dye method allows for *in vivo* imaging of depolarization of mitochondria in a live organism. This dye is also used *in vitro* to estimate the MMP (Misko et al., 2012). In addition, there are other near-infrared mitochondria probes for measuring the MMP (Ren et al., 2016). While live mitochondrial imaging demonstrates what is occurring in cells in real time, fixed mitochondrial imaging can also be performed. Mitotracker Red has been used to measure MMP in fixed tissues throughout the CNS (Qi et al., 2006). The advantage is that it stains mitochondria in live cells and the dye is well-retained after aldehyde fixation.

One relatively new assay used to measure mitochondria function in primary cells, established cell lines, spheroids, isolated mitochondria, and small tissue pieces is the Agilent Seahorse analyzer. The Agilent Seahorse Analyzer measures mitochondrial respiration and glycolysis in addition to the ATP production rate of live cells in a miniplate format. This analyzer allows for the measurement of oxygen consumption rate (OCR) and extracellular acidification rate (ECAR). OCR is proportional to mitochondrial respiration, while ECAR is proportional to glycolysis, and these allow measurement of mitochondrial activity. In addition to measuring basal respiration, the sensor cartridge contains ports to inject modulators into the cell wells during the assay to understand more aspects of ETC function. Most of the studies have been in cultured dorsal root ganglion cells, OLs, astrocytes, and neurons (Licht-Mayer et al., 2020; Apicco et al., 2021; Demaré et al., 2021). One of the first studies using ECAR/OCR studies in mice after 6 weeks of 0.3% CPZ diet showed significant changes in maximal respiration from cortical mitochondria (Singhal et al., 2020). The study was not complete, as a saturating response of oligomycin (an ATP Synthase inhibitor) was not shown. For a true comparison and assessment, a maximal response is required. Djordjevic et al. used the mitochondria stress test in a mouse model of PD (Djordjevic et al., 2020). This test can be applied to mouse models of MS as well to determine what kind of mitochondria dysfunction might occur in these models at specific timepoints. The mitochondria stress test can also be utilized using isolated mitochondria after induction of EAE or other mouse models of MS to regionally determine mitochondria dynamics at a specific timepoint after disease induction.

Another method to measure mitochondrial function is with an ATP assay to measure ATP output from cells. This has been performed in OLs (Rone et al., 2016; Chamberlain et al., 2021), and isolated optic nerve tissue (Yin et al., 2016). Another group designed an *in vivo* approach to understanding mitochondria function using an H_2_O_2_-sensitive GFP (roGFP-Orp1) and a fluorescent ATP sensor (ATeam) that targets mitochondria. These were used to image and measure the dynamics of ATP and H_2_O_2_ production in mitochondria of myelinated axons in the peripheral nervous system (PNS) (Imamura et al., 2009; van Hameren et al., 2019). While this technique was utilized in the PNS, this method could be adapted to understand mitochondria dynamics in the CNS.

### Cell-specific tagging of mitochondria

There is a pressing need to extend mitochondrial studies to tissues, particularly where cell culture-based models are inadequate in recapitulating complex cellular interactions, such as in MS. Currently, there are several mouse lines available through Jackson Laboratory that allow for studying mitochondria using CNS tissue specific Cre- expression.

One group developed a set of tools to visualize the dynamics of neuronal mitochondria by using mitochondrially targeted cyan (mitoCFP) and yellow (mitoYFP) fluorescent proteins selectively expressed in neurons under the control of Thy1 or *nse* (*Eno2)* regulatory elements, to make Thy1-mitoCFP and nse-mitoYFP mice (Misgeld et al., 2007). These MitoMouse lines contain elevated levels of fluorescent proteins consistent with specific labeling of mitochondria in neurons (Mar et al., 2014; Hayes et al., 2019). The group that designed this line was able to perform time-lapse recordings to assess mitochondria transport in peripheral nerves and measure mitochondria transport after axonal injury.

One commercially available strain is MITO-TAG (Jackson #032290). This mouse line was developed by a group at Caltech and contains a hemagglutinin (HA)-tagged EGFP that localizes to the OMM (Bayraktar et al., 2019). These mice conditionally express the (HA)-tagged EGFP targeted to the *Rosa26* locus with an upstream *loxP-*flanked termination signal. If crossed with Cre drivers, tissue specific expression of the (HA)-tagged EGFP is possible. While this line has not been utilized yet in CNS tissues, this strain may allow for neuroscientists to elucidate mitochondria dynamics and function that occur in specific cell types during disease progression and treatment.

A recent development to study mitochondria dynamics *in vivo* is with the use of photo-activatable mitochondria (phAM mice) (Pham et al., 2012). This mouse line has the photo convertible protein Dendra2 targeted to the *Rosa26* locus with an upstream *loxP-*flanked termination signal (Chudakov et al., 2007; Pham et al., 2012). The group used a 405 nm laser to photoconvert a subpopulation of mitochondria from green to red fluorescence (Pham et al., 2012). This allows mitochondria to be evaluated in fixed and live tissues. In addition, the photo switchable properties of Dendra2 allow subsets of mitochondria to be precisely monitored within a dense mitochondria network. This line can be crossed with Cre drivers to measure expression in specific cell types, which will allow researchers to determine whether mitochondria are being trafficked locally to meet a specific demand, or whether mitochondria are undergoing fission or fusion (Pham et al., 2012). When the phAM mice were crossed to *Meox2-Cre* mice, allowing for ubiquitous expression, all organs exhibited mito-Dendra2 fluorescence (Pham et al., 2012). Specifically, in the CNS, they saw expression in Purkinje cells. This study also specifically used PCP2-Cre to observe mitochondria expression only in Purkinje cells and in Purkinje cells with mutant Mfn2 (Pham et al., 2012).

To study mitochondria morphology *in vivo*, a reporter mouse with cell type-specific expression of YFP targeted to the mitochondrial matrix was developed (Sterky et al., 2011). Upstream of the mito-YFP transgene, a flox-flanked stop cassette was placed. This allowed for ubiquitous expression of mito-YFP with normal function of the respiratory chain (Sterky et al., 2011). These mice also can be crossed to visualize mitochondria expression via CNS cell type specific Cre expression.

The mito-QC (quality control) mouse line allows for studying mitophagy and mitochondrial architecture *in vivo*. This transgenic line contains an mCherry-GFP tag fused to the mitochondrial targeting sequence of the OMM protein, FIS1. The GFP tag has a pH-sensitive fluorescent mitochondrial signature which allows for the assessment of mitophagy and mitochondrial architecture (McWilliams et al., 2016). Under steady-state conditions, the mitochondrial network fluoresces both red and green; however, upon mitophagy, mitochondria are delivered to lysosomes where mCherry fluorescence remains stable, but GFP fluorescence becomes quenched by the acidic microenvironment, resulting in punctate mCherry-only foci (McWilliams et al., 2016; McWilliams and Ganley, 2019). Recently, to investigate spatio-temporal pattern of oxidative damage and dysfunction of OL mitochondria during inflammation, MOG-cre mice were crossed with cre-inducible mito-roGFP2-Orp1 and cre-inducible tdTomato reporter (Ai14) mice (Steudler et al., 2022). The resulting MOG-cre x mito-roGFP2-Orp1 x Ai14 model, GFP^+^ mitochondria could be detected in the tdTomato^+^ OL cell body and cytoplasmic extensions. After EAE induction, spinal cords were imaged using 2-photon and mitochondrial oxidation was quantified in OLs. Redox changes along with a significant impairment in organelle density and morphology were observed before and after clinical EAE development, specifically in OLs close to inflammatory immune cell containing lesions. Mitochondria oxidation levels reached near normal control levels during chronic EAE, however, mitochondria maintained altered morphology (Steudler et al., 2022).

Overall, recent advances in the development of various transgenic mitochondria reporter lines will advance cell specific understanding of mitochondria function in normal and diseased conditions.

## Conclusion

To clearly understand the role of mitochondria dysfunction in MS, requires an integrated clinical, biochemical, histological, and genetic approach. A major challenge has been a highly variable clinical and pathological feature of the disease. For the last few decades, mitochondrial studies were initiated in postmortem MS tissue or regions of tissue which lack the full picture of mitochondrial involvement in homeostatic conditions or neurodegenerations. Recently, progress has been made with assessing mitochondria related signaling pathways in biofluids of MS patients. However, the clear picture of mitochondria involvement in different cell types and how they contribute to MS disease initiation and progression is still lacking. For these reasons, animal model studies are necessary.

Primary brain cell cultures, organotypic brain slices and the availability of various mouse models of MS have indicated the role of mitochondria in disease progression. Improved techniques to assess mitochondrial genes and proteins along with spatial profiling along with assessing isolated mitochondria functional activity by extracellular flux analysis will further help us understand the clear role of mitochondria in initiation of the disease. The information obtained has been both tissue and cell culture based and does not mimic the complex environment in the brain. Thus, using cell specific genetically engineered reporter mitochondria mouse strains and using the aforementioned techniques will allow researchers to understand mitochondria function in different cell types in various mouse models of MS and extend the role of cell-specific mitochondria to MS pathophysiology.

## Author contributions

KA wrote much of the article and generated the figures. MO updated the references and tables. ST-W wrote, edited, and expanded the article and updated the figures. All authors contributed to the article and approved the submitted version.

## Funding

ST-W was the Principal Investigator in grants that were funded from National Multiple Sclerosis Society RG-2110-38560; RG-1901-33349 and National Institute of Health R01NS111552.

## Conflict of interest

The authors declare that the research was conducted in the absence of any commercial or financial relationships that could be construed as a potential conflict of interest.

## Publisher’s note

All claims expressed in this article are solely those of the authors and do not necessarily represent those of their affiliated organizations, or those of the publisher, the editors and the reviewers. Any product that may be evaluated in this article, or claim that may be made by its manufacturer, is not guaranteed or endorsed by the publisher.

## Figures and Tables

**Table:** 

Adenosine triphosphate	(ATP)
Pro-autophagy factor	(AMBRA1)
Autophagy related	(Atg)
B-cell leukemia/lymphoma 2	(BCL2)
BCL2 Interacting Protein 3	(BNIP3)
Cardiolipin	(CL)
Central nervous system	(CNS)
Cerebrospinal fluid	(CSF)
Cuprizone	(CPZ)
Cytochrome c oxidase	(COX)
Dynamin-related protein 1, Complex I	(NADH dehydrogenase)
Complex II	(succinate dehydrogenase)
Complex III	(ubiquinone cytochrome c oxidoreductase)
Complex I	(cytochrome c oxidase)
Complex V	(ATP synthase)
Deoxyribonucleic acid	(DNA)
Days post induction	(dpi)
Dynamin related protein 1	(Drp1)
Dynamin 2	(Dnm2)
Electron transport chain	(ETC)
Electron microscopy	(EM)
Experimental autoimmune encephalomyelitis	(EAE)
Flavin adenine dinucleotide	(FAD)
Carbonyl cyanide-4 (trifluoromethoxy) phenylhydrazone	(FCCP)
FUN14 Domain Containing 1	(FUNDC1)
γ-Aminobutyric acid	(GABA)
GABA type A receptor-associated protein	GABARAP
Guanosine triphosphate	(GTP)
GTPase effector domain	(GED)
Immunohistochemistry	(IHC)
Inner mitochondrial membrane	(IMM)
Huntingtin	(Htt)
Multiple sclerosis	(MS)
Mitochondrial	(mt)
Mitochondria membrane potential	(MMP; ΔΨ_m_)
Mitofusin 1 and 2	(Mfn1, Mfn2)
Mitochondrial fission factors	(Mff)
Microtubule-associated protein 1A/1B-light chain 3	(LC3)
Neighbor of BRCA1 gene 1 protein	(NBR1)
Calcium-binding and coiled-coil domain-containing protein 2	(NDP52)
Nicotinic adenine dinucleotide	(NAD)
Normal appearing white matter	(NAWM)
Ligand of BNIP3L	(NIX)
Optic atrophy 1	(OPA1)
Oligodendrocytes	(OLs)
Oligodendrocyte progenitor cells	(OPCs)
Optic neuropathy inducing” protein	(OPTN)
Primary mitochondrial oxidative phosphorylation	(OXPHOS)
Outer mitochondrial membrane	(OMM)
Peripheral blood mononuclear cells	(PBMCs)
Polymerase chain reaction	(PCR)
Primary progressive	(PP)
Peroxisome proliferator-activated receptor gamma coactivator 1	(Ppargc1α)
Peroxisome proliferator-activated receptor-gamma coactivator	(PGC)
Proteolipid protein	(PLP)
Photo-activatable mitochondria	(phAM)
Peripheral nervous system	(PNS)
Prohibitin 2	(PHB2)
Reactive oxygen species	(ROS)
Relapsing remitting multiple sclerosis	(RRMS)
Secondary progressive	(SP)
Shiverer	(*Shi*)
Syntaphilin	(snph)
Solute carrier family 25 member	(SLC25A)
Surface-Enhanced Laser Desorption Ionization Time of Flight Mass Spectrometry	(SELDI-TOF-MS)
Tricarboxylic acid	(TCA)
Translocase of the outer mitochondrial membrane complex subunit 20	(TOMM20)
Tax1-binding protein 1 is a protein	(TAX1BP1)
Tetramethylrhodamine methyl ester	(TMRM)
The class III phosphoinositide 3-kinase	(Vps34)
Voltage-dependent anion channel	(VDAC)
